# First study of correlation between oleic acid content and *SAD* gene polymorphism in olive oil samples through statistical and bayesian modeling analyses

**DOI:** 10.1186/s12944-018-0715-7

**Published:** 2018-04-10

**Authors:** Rayda Ben Ayed, Karim Ennouri, Sezai Ercişli, Hajer Ben Hlima, Mohsen Hanana, Slim Smaoui, Ahmed Rebai, Fabienne Moreau

**Affiliations:** 1 Laboratory of Molecular and Cellular Screening Processes, Genomics and Bioinformatics Group, Centre of Biotechnology of Sfax, PB ‘1177’, 3018 Sfax, Tunisia; 2 Department of Horticulture, Agricultural Faculty, Ataturk University, Erzurum, Turkey; 3 Unité de Biotechnologie des Algues, Biological Engineering Department, National School of Engineers of Sfax, University of Sfax, Sfax, Tunisia; 4 Laboratory of Extrêmophile Plants, Biotechnology Center of Borj-Cédria, B.P. 901, 2050 Hammam Lif, Tunisia; 5 Laboratory of Microorganisms and Biomolecules, Center of Biotechnology of Sfax, PB 1177, 3018 Sfax, Tunisia; 6 National Institute of Agricultural Research (INRA), Montpellier SupAgro, France

**Keywords:** Virgin olive oil, SNP, Fatty acid, Oleic acid, *SAD*

## Abstract

**Background:**

Virgin olive oil is appreciated for its particular aroma and taste and is recognized worldwide for its nutritional value and health benefits. The olive oil contains a vast range of healthy compounds such as monounsaturated free fatty acids, especially, oleic acid. The SAD.1 polymorphism localized in the *Stearoyl-acyl carrier protein desaturase* gene (*SAD*) was genotyped and showed that it is associated with the oleic acid composition of olive oil samples. However, the effect of polymorphisms in fatty acid-related genes on olive oil monounsaturated and saturated fatty acids distribution in the Tunisian olive oil varieties is not understood.

**Methods:**

Seventeen Tunisian olive-tree varieties were selected for fatty acid content analysis by gas chromatography. The association of SAD.1 genotypes with the fatty acids composition was studied by statistical and Bayesian modeling analyses.

**Results:**

Fatty acid content analysis showed interestingly that some Tunisian virgin olive oil varieties could be classified as a functional food and nutraceuticals due to their particular richness in oleic acid. In fact, the TT-SAD.1 genotype was found to be associated with a higher proportion of mono-unsaturated fatty acids (MUFA), mainly oleic acid (C18:1) (*r* = − 0.79, *p* < 0.000) as well as lower proportion of palmitic acid (C16:0) (*r* = 0.51, *p* = 0.037), making varieties with this genotype (i.e. Zarrazi and Tounsi) producing more monounsaturated oleic acid (C18: 1) than saturated acid. These varieties could be thus used as nutraceuticals and functional food.

**Conclusion:**

The SAD.1 association with the oleic acid composition of olive oil was identified among the studied varieties. This correlation fluctuated between studied varieties, which might elucidate variability in lipidic composition among them and therefore reflecting genetic diversity through differences in gene expression and biochemical pathways. *SAD* locus would represent an excellent marker for identifying interesting amongst virgin olive oil lipidic composition.

## Background

Olive oil could be defined as oil obtained exclusively from the olive fruit, whereas virgin olive oil, even also obtained from the fruit, is the one especially extracted by physical or mechanical techniques under specific conditions that do not lead to degradations, and which have not undergone any manipulation other than washing, decantation, centrifugation and filtration [1]. From ancient times and for several centuries, olive oil has been used for nutritional, medical, cosmetic, and other aims [2]. Actually, it constitutes one of the most important sources of fat and the principal one in Mediterranean diet associated with several healthy benefits [3]. This diet is characterized by a reasonably high intake of fruits, vegetables, fish, olive oil, nuts and a limited intake of saturated fat. Tur et al. [4] deduced that Mediterranean diet supplies a better health and quality of life for people who choose it. In addition, olive oil can be considered as an indispensable ingredient of the Mediterranean diet and implies that it may certainly have healthy benefits including reduction of coronary heart disease risks and prevention of several types of cancer [5].

Also, olive oil is known for its high levels of monounsaturated fatty acids (MUFA) and phenolic compounds: the major fraction, known as glyceride fraction, constituting approximatively 98% of oil’s weight and mostly composed of triacylglycerols, while some free fatty acids, monoglycerols and diglycerols can also be found. The typical fatty acid profile of virgin olive oil is made of oleic acid (65 to 85%) which is the main compound and classifies it among MUFA oils, as well as other fatty acids such as linoleic, palmitic and stearic acids [6]. Moreover, a minor fraction of olive oil presents almost 2% of its total weight and contains different components such as non-glyceride esters, aliphatic and triterpenic alcohols, sterols, hydrocarbons, polar pigments, tocopherols, phenolic compounds and volatiles [6]. Nevertheless, only a small number of these categories were identified as bioactive and are along with their benefits studied by Covas et al. [7]. Among other biological properties, olive oil compounds have been show to be efficient in decreasing the intensity of DNA oxidation damage [8, 9]. These studies have improved the importance in the promotion of health properties of olive oil.

Metabolic pathways of fatty acids biosynthesis involve malonyl acyl carrier protein (ACP) which is structured from the malonyl-CoA produced by ACCase, through a biological reaction catalyzed by malonyl-CoA: ACP transacylase. Fatty acids are then produced by a dissociable complex consisting of monofunctional enzymes and transferred to as fatty acid synthase. The enzymatic complex comprises six enzymes as well as the ACP, which combines the intermediate acyl chains [10]: β -ketoacyl-ACP synthases I, II, and III, β-ketoacyl-ACP reductase, β-hydroxyacyl-ACP dehydrase and enoyl-ACP reductase.

Being a key enzyme in the MUFA synthesis, our work aims to study the association between SNP localized in the *Stearoyl-acyl carrier protein desaturase SAD.1* gene and oleic acid content of Tunisian olive oil samples. Indeed, the *SAD* gene is responsible for the ubiquitous desaturation of C18:0 to C18:1, monounsaturated oleic acid intermediates [11]. Particularly, this study aims to evaluate this SNP and its association with oleic acid content and to identify SNPs usefulness in the quality characterization of Tunisian olive oils and consequently to its nutritional and healthy values.

## Methods

### Plant material

A total of 17 Tunisian olive-tree varieties were chosen from different geographical regions of Tunisia from north to south (Chetoui, Gerboui, Tounsi, Meski, Oueslati, El Horr, Chemlali Sfax, Chemlali Ontha, Chemlali Tataouine, Fakhari, Zalmati, Zarrazi, Chemchali, Besbessi, Fougi, Toffahi, Jemri Ben Guerdane). For each variety, leaves from two different trees were sampled for DNA analysis.

### DNA isolation

The DNA was extracted from leaves using the CTAB method described by Ben Ayed et al. [12] with and additional purification step, consisting in washing and eluting once with the QIAamp DNA stool (Qiagen) to eliminate contaminant compounds and generate a high quality DNA for specific, reproducible and consistent PCR amplifications [12]. Genomic DNA was dissolved in TE buffer (10 mM Tris–HCl pH 8.1 mM EDTA pH 8) and stored at − 20 °C until use.

### SNP genotyping

One SNP was selected within the *Stearoyl-acyl carrier protein desaturase* locus responsible for the ubiquitous desaturation of C18:0 to C18:1 FA intermediates. This SNP was genotyped by a polymerase chain reaction-restriction fragment length polymorphism (PCR-RFLP) method (Table 1). The PCR product (330 bp) of SNP (SAD1) was digested by *TaqI* restriction enzyme (Vivantis) at 65 °C for 16 h. This restriction enzyme recognizes the sequence CC/TT. The C-allele carrying PCR product was cleaved twice by the enzyme producing four fragments (263, 158, 105 and 67 bp). All digested products were separated by electrophoresis on 3% Nusieve ethidium bromide-stained agarose gels and visualized under UV light.

**Table 1:** Characteristics of SAD.1 SNP marker

Gene name	SNP code	Primer sequence (5′ → 3′)^a^	Tm ^b^	PIC	AF	GF
*Stearoyl-acyl carrier protein desaturase*	SAD.1(T/C)	5’gcaatatgaaagctccacat3’ 5’gtgccattgcgcatagcaaa3’	57 °C	0.439	*T* = 0.676 C = 0.323	TT = 0.470 CT = 0.411 CC = 0.117

*PIC* Polymorphism Content Information, *AF* Allele frequencies, *GF* Genotype frequencies

^a^Primers designed in this study

^b^annealing temperature for PCR amplification

### Olive oil extraction

The olive oil samples were obtained from fully ripened olives coming from various dual purpose and table Tunisian olive varieties. After harvesting, the olive fruit samples were immediately transported to the laboratory. Olive oil is produced by grinding 2.5 Kg stoned olives and extracted by mechanical means. The procedure for monovarietal oil production followed the standard methods used in oil factories, including milling, mixing for 30 min at 25 °C, centrifugation at 2000 g for 3 min and olive oil was obtained by natural decantation. Samples were stored into dark glass bottles at 4 °C until fatty acids composition analysis.

### Fatty acids composition analysis

The fatty acid methyl esters (FAMEs) were prepared as described by European Union standard methods (Commission Regulation (EEC) no. 2568/91). FAMEs were prepared by vigorously shaking a solution of oil in hexane (0.2 g in 3 mL) with 0.4 mL of 2 N methanolic potassium hydroxide, and analyzed by gas chromatography with a Shimadzu chromatograph equipped with a flame ionization detector (FID), and a fused silica column (30 m length × 0.32 mm i.d. and thickness of 0.25 μm, formed with 50% cyanopropylmethyl- 50% phenylmethyl-polysiloxane). An injection volume of 1 μl was used. The carrier gas was nitrogen with a flow rate of 1 mL/min. The injector and detector temperatures were set at 220 °C, whereas the oven temperature was held at 180 °C. Seven fatty acids including palmitic (C_16:0_), palmitoleic (C_16:1_), stearic (C_18:0_), oleic (C_18:1_), linoleic (C_18:2_), linolenic (C_18:3_) and arachidic (C_20:0_) acids were identified from their retention times.

### Statistical analysis

The analysis of the relationship between SAD.1 SNP marker and the fatty acids composition was performed in many steps using several statistical techniques.

For Fatty acids composition, the t- test or one-way analysis of variance (one-way ANOVA) was used to assess the significant difference between the means of genotype groups for this SNP.

The Pearson’s correlation analysis was used to test associations between variables. All analyses were performed using R program. Two-sided *P*-values< 0.05 were considered statistically significant. Moreover, R language was used to draw the Directed Acyclic Graph (DAG), using the ‘growshrink’ algorithm. The algorithm efficiently filters links out of a full skeletal DAG, in which all nodes are primarily connected (except those having no relationships with others), based on tests of conditional independence between a pair of nodes given all possible subsets of the rest. Logical rules are applied to establish the direction of links (conditional dependence between variables), so that cycles are not introduced and patterns of conditional independence found in the data match the generated DAG. We estimated link influence in the final DAG by calculating the regression beta-coefficient for each potential causal effect in which the variable at the base of the arrow (‘cause’) was considered a covariate, and the variable at the head of the arrow (‘effect’) was considered the outcome or dependent variable. The advantage of Bayesian network is to deduce all parent nodes which are directly dependent on child nodes [13].

## Results

### Characteristics of the studied SNP markers

In the present study, PIC value observed in olive varieties for SAD.1 marker was 0.439. The SAD.1 SNP marker appeared to be a polymorphic marker. In fact, this result demonstrates that SAD.1 is an informative marker and able to distinguish between studied olive oils.

The allelic frequencies of the studied SNP showed that there is a dominance of the T allele (67.6%). Most of the studied varieties have heterozygous genotypes (Table 1) or homozygous TT. However, the frequency of CC-SAD.1 genotype is about 11.7%.

### Genotypic associations of SAD.1 SNP with fatty acid composition by using bivariate and multivariate statistical analyses

It has been reported that *SAD* gene was implicated in the transformation of the saturated stearic fatty acid C18:0 to the monounsaturated oleic fatty acid C18:1, therefore, we analyzed the association of SAD.1 (C/T) polymorphism and the fatty acids composition of each olive oil sample (Table 2).

**Table 2:** Fatty acids composition in the studied olive oil varieties

	SFA	IFA	MUFA	PUFA	C16:0	C16:1	C18:0	C18:1	C18:2	C18:3	C20:0
Zarrazi	13,39 ± 0,27	89,61 ± 2,69	78,28 ± 3,13	12,36 ± 0,12	10,30 ± 0,21	0,82 ± 0,02	2,32 ± 0,09	75,19 ± 0,75	13,39 ± 0,2	0,59 ± 0,02	0,41 ± 0,02
Chetoui	15,45 ± 0,31	87,55 ± 2,63	70,04 ± 2,8	18,54 ± 0,19	13,13 ± 0,26	0,52 ± 0,02	1,96 ± 0,08	68,50 ± 0,68	17,51 ± 0,3	0,67 ± 0,01	0,39 ± 0,01
Chemchali	18,54 ± 0,37	84,46 ± 2,53	72,10 ± 2,88	13,39 ± 0,13	15,97 ± 0,32	1,96 ± 0,03	2,16 ± 0,09	67,98 ± 0,55	14,16 ± 0,2	0,46 ± 0,01	0,31 ± 0,01
Oueslati	13,39 ± 0,27	89,61 ± 2,69	77,25 ± 3,09	11,33 ± 0,11	10,82 ± 0,22	0,88 ± 0,03	2,06 ± 0,08	75,71 ± 0,77	12,36 ± 0,5	0,57 ± 0,03	0,41 ± 0,02
El Horr	18,54 ± 0,37	84,15 ± 2,52	74,16 ± 2,97	9,99 ± 0,1	15,97 ± 0,3	2,06 ± 0,03	1,18 ± 0,05	72,62 ± 0,7	10,30 ± 0,4	0,57 ± 0,05	0,28 ± 0,02
Toffahi	17,51 ± 0,35	85,08 ± 2,55	70,04 ± 2,8	15,04 ± 0,15	14,68 ± 0,25	2,01 ± 0,06	2,06 ± 0,08	69,01 ± 0,5	14,16 ± 0,55	0,57 ± 0,04	0,36 ± 0,04
Fakhari	16,48 ± 0,33	86,52 ± 2,6	75,19 ± 3,01	11,85 ± 0,12	15,97 ± 0,31	1,29 ± 0,04	3,66 ± 0,15	69,27 ± 0,9	11,54 ± 0,25	0,55 ± 0,05	0,65 ± 0,03
Fougi	19,57 ± 0,39	83,43 ± 2,5	69,01 ± 2,76	14,42 ± 0,14	15,45 ± 0,3	1,91 ± 0,06	1,39 ± 0,06	66,95 ± 0,66	15,97 ± 0,32	0,78 ± 0,05	0,46 ± 0,02
Meski	19,57 ± 0,39	83,43 ± 2,5	69,01 ± 2,76	14,42 ± 0,14	17,51 ± 0,34	0,67 ± 0,02	1,60 ± 0,06	53,56 ± 0,5	27,30 ± 0,55	0,98 ± 0,03	0,28 ± 0,01
Tounsi	16,48 ± 0,33	86,31 ± 2,5	78,28 ± 3,13	8,03 ± 0,08	13,13 ± 0,26	1,13 ± 0,03	2,16 ± 0,09	77,25 ± 0,5	7,83 ± 0,16	0,62 ± 0,02	0,38 ± 0,01
Besbessi	19,57 ± 0,39	83,33 ± 2,5	67,98 ± 2,72	15,45 ± 0,15	17,87 ± 0,39	1,91 ± 0,06	1,49 ± 0,05	65,41 ± 0,6	14,94 ± 0,3	0,80 ± 0,01	0,31 ± 0,02
Chemlali Sfax	21,63 ± 0,43	81,16 ± 2,43	61,80 ± 2,47	19,57 ± 0,2	19,57 ± 0,39	2,68 ± 0,08	1,98 ± 0,07	58,71 ± 0,59	19,57 ± 0,39	0,72 ± 0,02	0,31 ± 0,02
Chemlali tataouine	20,60 ± 0,41	81,99 ± 2,46	73,13 ± 2,93	8,65 ± 0,09	16,48 ± 0,33	2,58 ± 0,09	2,37 ± 0,1	70,04 ± 0,7	10,30 ± 0,21	0,62 ± 0,02	0,31 ± 0,03
Zalmati	22,66 ± 0,45	80,34 ± 2,41	61,80 ± 2,47	18,54 ± 0,19	19,88 ± 0,4	2,37 ± 0,07	2,27 ± 0,12	59,43 ± 0,6	17,82 ± 0,36	0,58 ± 0,03	0,32 ± 0,02
Chemlali ontha	18,54 ± 0,37	84,46 ± 2,53	73,13 ± 2,93	11,33 ± 0,11	15,97 ± 0,32	2,58 ± 0,08	2,37 ± 0,08	70,25 ± 0,7	10,61 ± 0,2	0,62 ± 0,05	0,31 ± 0,01
Gerboui	15,45 ± 0,31	87,55 ± 2,63	66,95 ± 2,68	21,63 ± 0,22	13,18 ± 0,26	0,82 ± 0,02	1,65 ± 0,07	64,99 ± 0,65	20,50 ± 0,51	0,72 ± 0,07	0,31 ± 0,01
Jemri Ben Guerdane	22,66 ± 0,45	80,34 ± 2,41	66,95 ± 2,68	13,39 ± 0,13	20,50 ± 0,41	2,24 ± 0,07	1,90 ± 0,09	64,68 ± 0,65	12,36 ± 0,25	0,82 ± 0,06	0,33 ± 0,01

*SFA* saturated fatty acids, *IFA* Insaturated fatty acids, *MUFA* monounsaturated fatty acids, *PUFA* polyunsaturated fatty acids, *C16:0* Palmitic acid, *C16:1* Palmitoleic acid, *C18:0* Stearic acid, *C18:1* Oleic acid, *C18:2* Linoleic acid, *C18:3* Linolenic acid, *C20:0* Arachidic acid

The analysis of SAD.1 marker revealed three genotypes for this SNP: CT, TT and CC. 41.1% of the varieties were heterozygous CT-SAD.1.

Table 3 shows results of *p*-values generated by the variance analysis. No significant associations were showed between SAD.1 SNP marker and the other parameters. However, highly significant association of this marker with the accumulation of oleic monounsaturated fatty acid is proved. In fact, highly association was established with the accumulation of the oleic monounsaturated fatty acid (*P*_*Duncan*_ = < 0.000, *P*_*Tukey*_ = 0.003, *P*_*Scheffe*_ = 0.007) by using Duncan, Tukey and Scheffe post-hoc tests, respectively. The homozygous varieties TT-SAD.1 (Chemlali Tataouine, Oueslati, Zarrazi and Tounsi) have a higher oleic acid level than the heterozygous varieties CT (such as Chetoui and Besbessi) and the homozygous varieties CC (Meski, Chemlali Sfax and Zalmati). Interestingly, we found that the rate of oleic acid was significantly lower in the varieties carrying the homozygous genotype CC-SAD.1 (especially the oil purpose varieties like Chemlali Sfax (56.2%) and Zalmati (53.1%) and the table olive variety Meski (51.3%)) than the homozygous genotypes (TT). Moreover, CT varieties have a medium oleic fatty acid rate than the two homozygous genotypes CC and TT.

**Table 3:** The association between SAD.1 SNP and Fatty acid compositions using ANOVA test

Parameters	SNP	SAD.1			*P*_*Duncan*_	*P*_*Tukey*_	*P*_*Scheffe*_
		TT	CT	CC			
SFA	Mean ± SD	16.375 ± 0.914	18.571 ± 0.977	20 ± 1.828	0.083	0.159	0.183
IFA	Mean ± SD	83.462 ± 0.938	81.414 ± 1.003	79.900 ± 1.876	0.096	0.182	0.208
MUFA	Mean ± SD	72.750 ± 1.113	65.857 ± 1.190	63.500 ± 2.227	0.313	0.561	0.590
PUFA	Mean ± SD	10.750 ± 0.949	16.000 ± 1.015	16.500 ± 1.898	0.798	0.963	0.967
C16:0	Mean ± SD	13.750 ± 0.907	16.086 ± 0.969	18 ± 1.813	0.224	0.086	0.103
C16:1	Mean ± SD	1.619 ± .271	1.624 ± .290	1.625 ± 0.543	0.992	0.990	0.997
C18:0	Mean ± SD	2.206 ± .182	1.777 ± .195	1.735 ± .365	0.246	0.431	0.463
C18:1	Mean ± SD	70.306 ± 1.093	63.514 ± 1.169	54.500 ± 2.186	**< 0.000**	**0.003**	**0.007**
C18:2	Mean ± SD	10.981 ± 0.924	15.707 ± 0.988	22.750 ± 1.848	0.051	0.059	0.072
C18:3	Mean ± SD	0.570 ± 0.035	0.671 ± 0.037	0.825 ± 0.070	0.171	0.347	0.379
C20:0	Mean ± SD	0.377 ± 0.031	0.337 ± 0.033	0.287 ± 0.062	0.195	0.353	0.385

Bold values: each variable that has statistical significance for all tests was declared when *P*-values are < 0.05;

*P* *P*-value of ANOVA analysis, *SD* standard deviation

### Genotypic associations of SAD.1 SNP with fatty acid composition by using Bayesian networks modeling

Bayesian networks modeling was used and applied in order to better understand and highlight the relationship between the molecular marker SAD.1 and fatty acid composition of the studied olive oil varieties.

**Fig. 1:**
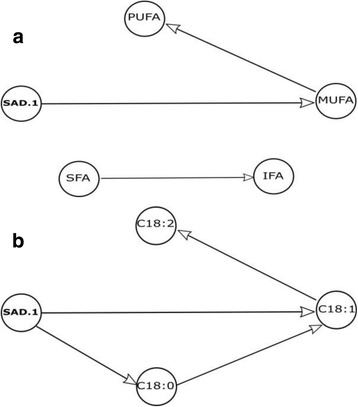
Directed acyclic graph representing possible SAD.1SNP marker connections with SFA, IFA, MUFA and PUFA (**a**), and with stearic (C18:0), oleic (C18:1), linoleic (C18:2) and linolenic (C18:3) fatty acid (**b**)

Firstly, we considered 5 nodes as represented in Fig. 1a. Correlation coefficients among fatty acid compositions in olive oil varieties are presented in Table 4. MUFAs, particularly oleic acid, are responsible for the most important nutritional and healthy properties of olive oil [14]. In our study, based on the molecular marker “SAD.1” that has one connection, “SAD.1” is negatively correlated with MUFAs (*r* = − 0.79; *p* < 0.000) and on the other hand, positively correlated with PUFAs (*r* = 0.74; *p* = 0.001).

**Table 4:** Pearson’s correlations of SAD.1 marker with fatty acid compositions of the studied olive oil cultivars

Parameters	SAD.1	
	*r*	*p*
SFA	0.474	0.054
IFA	−0.446	0.073
MUFA	−0.790	**< 0.000**
PUFS	0.740	**0.001**
C16:0	0.510	**0.037**
C16:1	0.004	0.988
C18:0	− 0.424	0.090
C18:1	−0.773	**< 0.000**
C18:2	0.729	**0.001**
C18:3	0.580	**0.015**
C20:0	−0.311	0.224

Bold values: each variable that has statistical significance was declared when *P*-values are < 0.05

*P* *P*-value, *r* correlation coefficient

Moreover, Fig. 1b shows that the molecular marker “SAD.1” was negatively influenced by the saturated stearic acid C18:0 (*r* = − 0.507; *p* = 0.04) and the monounsaturated oleic acid C18:1 (*r* = − 0.773; *p* < 0.000). Furthermore “SAD.1” node was positively influenced by the polyunsatured linoleic acid C18:2 (*r* = 0.729; *p* = 0.001) and linolenic acid C18:3 (*r* = 0.580; *p* = 0.015). The linoleic acid is directly influenced by oleic acid. Besides, stearic and oleic acids are directly influenced by the SAD.1 marker. SAD.1 markers play a key role in the fatty acids composition of each olive oil varieties. This finding could be explained by the fact that SAD.1 SNP is located within a gene involved in the process of synthesis of the oleic acid [15], suggesting the direct effect of the SAD.1 genotype variations on the percentage of mono-unsaturated fatty acid for each variety.

## Discussion

The consumption of virgin olive oil keeps being very important in the Mediterranean area and is increasing throughout the world due to its beneficial effects in diets and health. Indeed, virgin olive oil contains a huge range of healthy compounds such as mono-unsaturated free fatty acids (mainly oleic acid C18:1), phenolic compounds, squalene, tocopherols and sterols. Therefore, virgin olive oil can be used as a functional food or nutraceuticals because its consumption was associated with the prevention and therapy for many diseases including cardiovascular pathologies, dyslipidaemia, arthrosclerosis, osteoporosis, …. Nevertheless, the yield of the bioactive molecules containing in the virgin olive oil depends on climate, ripeness of olives oil extraction process and mainly on the variety.

Nonetheless, little is known about the important correlation between genotype and oleic acid variation among olive varieties. The present study, demonstrated that oleic acid amount is strictly linked to the SAD.1 SNP marker localized in the *SAD.1* gene which is involved in the process of synthesis of the monounsaturated oleic acid, particularly in the transformation of the saturated stearic acid C18:0 to the monounsaturated oleic acid C18:1.

In the current work, for each studied olive oil variety, we determined the fatty acids composition and the SNP (SAD.1) genotype. Subsequently, based on bivariate, multivariate and bayesian networks analysis, we confirmed the variation effect of this SNP on the fatty acids profile, especially, stearic and oleic fatty acids.

The findings showed that the stearic fatty acid C18:0 and the oleic fatty acid C18:1 levels were related to SAD.1 SNP genotypes. Indeed, this SNP was significantly associated with C18:0 and C18:1 proportions in the olive oil varieties. These results suggested that this locus may be a stearic and oleic acid specific SNP.

This correlation was proved by the statistical and modeling analyses used in this study. We showed that the homozygous genotype TT was positively correlated with the level of C18:1 and negatively correlated with the level of saturated fatty acid (SFA) level, especially C16:0. Besides, we demonstrated that the SAD.1 SNP genotype variations were significantly associated with the fatty acids levels. In fact, the homozygous SAD.1-CC genotype was negatively correlated, at a high significant level, with the C18:1 level (*r* = − 0.773, *p* < 0.000) and positively correlated with C16:0 level (*r* = 0.501, *P* = 0.037). This results concern essentially three varieties: Meski, Chemlali Sfax which had the genotype CC-SAD.1. Nonetheless, the heterozygous genotype CT was correlated with a moderate amount of oleic acid and stearic acid.

Previous research works [16, 17] reported that olive oil fatty acid composition, particularly oleic acid fluctuated according to the variety. However, until now, no work studied the genetic initial point of these fatty acid fluctuations. Accordingly, our present paper supports that the SAD.1 SNP might be a useful tool to explicate the genetic basis of oleic acid variations in olive oil varieties and it might be a predictive genotype marker to identify high quality of olive oil with high nutraceutical value. Moreover, alterations in this SNP caused changing oleic fatty acid levels among olive oil varieties, indicating that oleic acid content variations mirror genetic diversity, which may affect dissimilarity in many genetic (.i.e. gene expression, protein function,…) and environmental issues and their complicated connections. Consequently, our judgment might in part give explanation to this inconsistency.

Previous studies showed that virgin olive oil could be used in preventing and in such case treating dyslipidaemia. However, in order to use virgin olive oil as a functional and traditional nutraceutical food, it is required to select the best varieties which have high level in monounsaturated oleic acid.

However, up to this time the subject about the lipid-lowering properties of virgin olive oil is still under debate. Indeed, the course of action controlling this property is complex. Recently, Estruch and coworkers (2013) [18] studied the impact of the use of virgin olive oil as a food complement and they revealed that these nutraceuticals can decrease occurrence of main cardiovascular crisis provoked by dyslipidaemia. The accurate internal mechanisms causing such action are not entirely understood but may be related to several postulations. In effect, nutraceuticals events of virgin olive oil in lipid mechanism in human body can be act on numerous biochemical pathways able to control lipid disorder in cell.

Current studies explained that the nutraceuticals play a peculiar role in improving human dyslipidaemia and may represent valuable compounds in the supervision of lipid chaos, probably through decreasing of the secretion of very low-density lipoprotein, the reduction of 7α-hydrolase or reducing of the 3-hydroxy-3-methyl glutanyl-CoA reductase mRNA levels [19–22].

Consequently, a key strong point of our present paper is being the original and first report about the impact of SNP located in *SAD* gene on variability levels of oleic acid content in virgin olive oils. The association of SAD.1 SNP with saturated, mono and polyunsaturated fatty acid profiles of virgin olive oil varieties was assessed in the present study. Furthermore, we highlighted the effect of this SNP and we did explore relations effects between them. Indeed, the combination between molecular marker, statistical and modelling analysis used in this work may be considered as an effective and reliable tools to study the compositional quality of worldwide virgin olive oil and then to select the best varieties for nutraceutical use.

## Conclusions

To the best of our knowledge, this is the first work that shows and reveals that oleic acid, the main MUFA of olive oil, is correlated with the SAD.1 SNP located in the coding region of *SAD* gene. This correlation diverged among considered varieties, which might elucidate oleic acid content differences between varieties reflecting thus genetic diversity, as well as variability in gene regulation activity and metabolite pathways. Hence, this SNP marker could be useful and informative about the quality of olive oils and subsequently could advise the most excellent olive oil varieties for customers based on their SNP genotypes.

## Abbreviations

MUFA: Mono-unsaturated fatty acid
PUFA: Poly-unsaturated fatty acid
*SAD*: *Stearoyl-acyl carrier protein desaturase*
SFA: saturated fatty acids
SNP: Single nucleotide polymorphism
UFA: unsaturated fatty acids
